# Safety Monitoring of the Janssen (Johnson & Johnson) COVID-19 Vaccine — United States, March–April 2021

**DOI:** 10.15585/mmwr.mm7018e2

**Published:** 2021-05-07

**Authors:** David K. Shay, Julianne Gee, John R. Su, Tanya R. Myers, Paige Marquez, Ruiling Liu, Bicheng Zhang, Charles Licata, Thomas A. Clark, Tom T. Shimabukuro

**Affiliations:** 1 CDC COVID-19 Response Team.

On February 27, 2021, the Food and Drug Administration (FDA) issued an Emergency Use Authorization (EUA) for Janssen (Ad.26.COV2.S) COVID-19 vaccine (Janssen Biotech, Inc., a Janssen Pharmaceutical company, Johnson & Johnson) (*1*). The Janssen COVID-19 vaccine, the third COVID-19 vaccine authorized for use in the United States, uses a replication-incompetent human adenoviral type 26 vector platform* (*2*) and is administered as a single intramuscular dose, whereas the first two authorized vaccines use an mRNA platform and require 2 doses. On February 28, 2021, the Advisory Committee on Immunization Practices (ACIP) issued interim recommendations for use of Janssen COVID-19 vaccine among persons aged ≥18 years (*3*). During April 13–23, CDC and FDA recommended a pause in use of Janssen vaccine after reports of six cases of cerebral venous sinus thrombosis (CVST) with thrombocytopenia (platelet count <150,000/*μ*L of blood) among Janssen vaccine recipients (*4*). Similar thrombotic events, primarily among women aged <60 years, have been described in Europe after receipt of the AstraZeneca COVID-19 vaccine, which uses a replication-incompetent chimpanzee adenoviral vector (*5*–*7*). The U.S. CVST cases that prompted the pause in Janssen vaccination, as well as subsequently detected CVST cases, are described elsewhere (*8*). This report summarizes adverse events among Janssen vaccine recipients, including non-CVST cases of thrombosis with thrombocytopenia syndrome (TTS), reported to the Vaccine Adverse Events Reporting System (VAERS), a passive surveillance system, and through v-safe, an active monitoring system. As of April 21, 2021, 7.98 million doses of the Janssen COVID-19 vaccine had been administered. Among 13,725 VAERS reports reviewed, 97% were classified as nonserious and 3% as serious,^†^ including three reports among women of cases of thrombosis in large arteries or veins accompanied by thrombocytopenia during the second week after vaccination. These three cases and the previously detected CVST cases are consistent with 17 cases of TTS,^§^ a newly defined condition. Approximately 338,700 Janssen COVID-19 vaccine recipients completed at least one v-safe survey during the week after vaccination; 76% reported a systemic reaction, 61% reported a local reaction, and 34% reported a health impact.^¶^ Fatigue and pain were commonly reported symptoms in both VAERS and v-safe. The overall safety profile is consistent with preauthorization clinical trials data. Prompt review of U.S. vaccine safety data detected three additional cases of non-CVST TTS, in addition to the previously recognized CVST cases that initiated the pause in use of the Janssen COVID-19 vaccine. Ongoing monitoring of adverse events after COVID-19 vaccination, including vaccination with the Janssen single-dose vaccine, is essential for evaluating the risks and benefits of each vaccine.

VAERS is a national passive surveillance program managed by CDC and FDA that monitors adverse events after all vaccinations (*9*). VAERS reports are accepted from health care providers, vaccine manufacturers, and the public. Under EUAs for each COVID-19 vaccine, health care providers are required to report several types of adverse events to VAERS, including all deaths.** Signs and symptoms in VAERS reports are coded using the Medical Dictionary for Regulatory Activities (MedDRA).^††^ VAERS staff members attempt to obtain medical records and supporting information from health care providers for all reported serious events, as well as death certificates and autopsy reports for all deaths.

V-safe is a new, voluntary text-based surveillance system designed to collect additional information about COVID-19 vaccine adverse events, particularly for common side effects.^§§^ Vaccine recipients who enroll in v-safe receive regularly scheduled text message reminders to complete short online health surveys that include questions about local injection site and systemic reactions and health impacts (i.e., whether the enrollee was unable to perform normal daily activities, missed work, or received care from a medical professional because of new symptoms or conditions).^¶¶^ Enrollees who report seeking medical care are contacted by CDC’s v-safe call center and encouraged to complete a VAERS report, if indicated.

In this report, VAERS and v-safe data are described by sex, age group, and race/ethnicity of vaccine recipients. VAERS data include reports received and processed during March 2–April 21. V-safe data from persons vaccinated during March 2–April 12 were analyzed to permit time for respondents to complete up to eight daily health surveys after vaccination. These activities were reviewed by CDC and are consistent with applicable federal law and CDC policy.***

As of April 21, 2021, 7.98 million doses of Janssen COVID-19 vaccine had been administered in the United States, 50% to women. The median age at vaccination was 50 years. Race/ethnicity was unknown for 39% of persons vaccinated; 38% were non-Hispanic White (White).

## Review of VAERS Data

VAERS received and processed^†††^ 13,725 adverse event reports for Janssen COVID-19 vaccine recipients; median age was 42 years, and 66% were women (Table 1). Among these VAERS reports, 13,294 (97%) were classified as nonserious, and 343 (3%) were classified as serious, including three reports of non-CVST TTS (no deaths). Two of the TTS cases occurred among women aged 30–39 years and one in a woman aged 50–59 years. Each of these women had evidence of large-vessel thrombosis and thrombocytopenia (Table 2). As of April 25, 14 CVST cases had been confirmed (*8*), for a total of 17 TTS cases.

**TABLE 1:** Percentage of nonserious and serious adverse events after receipt of Janssen COVID-19 vaccine, by demographic characteristics of vaccine recipients and reported symptoms — Vaccine Adverse Event Reporting System, United States, March–April 2021

Characteristic	Total (N = 13,725)	Severity of adverse event, %*		
		Nonserious (n = 13,294)	Serious, excluding death (n = 343)	Death (n = 88)
**Sex**				
Female	**66.2**	66.6	57.1	50.0
Male	**31.2**	30.8	40.5	43.2
Unknown	**2.6**	2.6	2.3	6.8
**Age group, yrs**				
0–17	**1.5**	1.6	0.6	0.0
18–49	**57.0**	57.9	34.4	13.6
50–64	**26.8**	26.7	33.8	18.2
65–74	**6.8**	6.5	14.3	18.2
75–84	**1.8**	1.5	6.4	15.9
≥85	**0.6**	0.4	3.2	19.3
Unknown	**5.6**	5.5	7.3	14.8
**Race/Ethnicity**				
Hispanic or Latino	**7.3**	7.3	6.1	1.1
Non-Hispanic or Latino				
American Indian or Alaska Native	**0.2**	0.2	0.0	0.0
Asian	**2.3**	2.3	1.5	3.4
Black	**3.4**	3.3	5.5	8.0
Native Hawaiian or Pacific Islander	**0.1**	0.1	0.0	0.0
White	**58.4**	58.6	52.2	45.5
Multiracial	**1.3**	1.3	0.9	0.0
Other	**0.4**	0.4	0.3	1.1
Unknown race	**0.5**	0.5	1.5	2.3
Unknown ethnicity				
American Indian or Alaska Native	**0.1**	0.1	0.0	0.0
Asian	**0.3**	0.3	0.6	2.3
Black	**0.8**	0.7	2.6	1.1
Native Hawaiian or Pacific Islander	**<0.1**	<0.1	0.0	0.0
White	**5.8**	5.7	8.8	9.1
Multiracial	**0.2**	0.2	0.0	0.0
Other	**1.7**	1.7	0.9	0.0
Unknown race/ethnicity	**17.4**	17.3	19.2	26.1
**Reported symptoms**				
Headache	**34.4**	35.0	17.8	6.8
Fever	**33.7**	34.2	21.6	8.0
Chills	**32.7**	33.3	14.9	4.6
Pain	**25.5**	26.1	10.2	1.1
Fatigue	**23.9**	24.3	12.8	5.7

* Reports are classified as serious if any of the following are reported: death, life-threatening illness, hospitalization or prolongation of hospitalization, permanent disability, congenital anomaly, or birth defect.

**TABLE 2:** Characteristics of patients with evidence of thrombosis with thrombocytopenia syndrome* after receipt of Janssen COVID-19 vaccine — Vaccine Adverse Events Reporting System, United States, March–April, 2021

Patient	Age group, yrs	Days to symptom onset after vaccination	Initial signs and symptoms	Later signs and symptoms	Lowest platelet count^†^	Anti-PF4 antibody status^§^	Location of thrombus/occlusion
A	30–39	10	Headache, left-sided paresis	Headache, left-sided paresis	60,000/*μ*L	Positive	Right carotid artery, left brachial vein, right femoral vein
B	50–59	11	Left leg swelling, bruising	Bilateral lower extremity swelling	15,000/*μ*L	Not available	Left lower extremity deep vein, right femoral artery, left and right iliac arteries
C	30–39	6	Nausea, vomiting, shortness of breath, altered mental status	Nausea, vomiting, shortness of breath, altered mental status	20,000/*μ*L	Not available	Portal vein, superior mesenteric and splenic arteries, pulmonary artery

**Abbreviations:** PF4 = platelet factor 4; TTS = thrombosis with thrombocytopenia syndrome.

* Patients with evidence of TTS not classified as cerebral venous sinus thrombosis. Brighton Collaboration’s draft interim case finding definition for TTS: any patient presenting with acute venous or arterial thrombosis and new onset thrombocytopenia, with no known exposure to heparin or any other underlying condition or explanation for the condition. https://brightoncollaboration.us/wp-content/uploads/2021/04/TTS-Case-Finding-and-Definition-Process.v1.0-1-1.pdf

^†^ Normal range = 150,000–450,000/*μ*L.

^§^ The heparin:PF4 complex is the antigen in heparin-induced thrombocytopenia, an autoimmune reaction to administration of heparin, an anticoagulant. Anti-PF4 antibodies also have been found in patients with thrombosis who have no known exposure to heparin. Anti-PF4 antibodies have been detected in persons with thrombosis and thrombocytopenia after receipt of Janssen and AstraZeneca COVID-19 vaccines (Scully M, Singh D, Lown R, et al. Pathologic antibodies to platelet factor 4 after ChAdOx1 nCoV-19 vaccination. N Engl J Med 2021. Epub April 16, 2021).

CDC and FDA reviewed 88 reports of death after receipt of Janssen COVID-19 vaccine; death certificates were available for 12 (14%). Among the 88 reported decedents, 44 were female, 38 were male, and the sex of six was not reported (Table 1). The median decedent age was 69 years (range = 21–97 years); median interval from vaccination to death was 2 days (range = 0–23 days). All death reports received a medical review^§§§^; the most frequent preliminary impressions of CDC and FDA reviewers regarding cause of death were 1) decedent found dead, with no additional details available (34 reports); 2) cardiac arrest or cardiovascular disease (23 reports); 3) COVID-19 disease (eight reports); and 4) cerebrovascular disease (five reports). As of most recent follow-up (April 28, 2021), three patients with TTS had died. Among 79 reports of anaphylaxis after vaccination, four were confirmed as anaphylaxis cases after interview with a health care provider or review of medical records (<0.5 cases per 1 million doses administered); four reports remain under review. Headache (34%), fever (34%), chills (33%), injection site pain (26%), and fatigue (24%) were the symptoms most frequently reported to VAERS (Table 1).

## Review of v-safe Data

During March 2–April 12, v-safe enrolled 338,765 Janssen COVID-19 vaccine recipients who completed at least one postvaccination survey. The median age of v-safe enrollees was 46 years (range = 15–109 years); 60% were women (Supplementary Table, https://stacks.cdc.gov/view/cdc/105473). Sixty-seven percent of enrollees identified as White. During days 0–7 after vaccination, 76% of enrollees reported at least one systemic reaction, and 61% reported at least one injection site reaction (Table 3). Fatigue, pain, and headache were the most commonly reported reactions. Symptoms were most frequently reported on the first day after vaccination; the proportion of enrollees reporting specific reactions decreased with number of days since vaccination. On postvaccination day 1, 28% of enrollees reported being unable to perform normal, daily activities, and 16% reported being unable to work. Only 1.4% of enrollees reported seeking any form of medical care in the 7 days after vaccination.

**TABLE 3:** V-safe enrollees who completed at least one survey and reported a local or systemic reaction or health impact on days 0–7 after receiving Janssen COVID-19 vaccine — United States, March 2–April 12, 2021

Event	Percentage of enrollees reporting reaction or health impact								
	Days 0–7*	Day 0	Day 1	Day 2	Day 3	Day 4	Day 5	Day 6	Day 7
**Total enrollees, no. (%) of enrollees reporting^†^**	**338,765 (100)**	**207,483 (61)**	**259,535 (77)**	**261,096 (77)**	**251,676 (74)**	**238,946 (71)**	**225,427 (67)**	**209,958 (62)**	**202,138 (60)**
**Reaction reported**									
Fatigue	59.1	17.9	56.3	26.2	16.7	12.8	11.0	9.8	9.0
Injection site pain	57.9	31.6	48.5	39.1	30.1	21.5	13.7	7.9	5.0
Headache	52.2	13.0	50.8	19.9	10.8	8.1	7.6	7.5	7.4
Myalgia	47.8	9.0	47.9	19.2	9.7	6.6	5.3	4.6	4.4
Fever	34.7	4.8	37.0	8.3	2.8	1.8	1.4	1.2	1.2
Chills	34.2	5.5	35.7	6.7	2.3	1.4	1.2	1.0	1.0
Joint pain	26.1	3.5	25.3	8.9	4.5	3.3	2.8	2.6	2.4
Nausea	18.7	3.8	15.7	5.4	3.5	2.6	2.1	1.9	1.7
Diarrhea	9.4	0.9	4.3	3.4	2.7	2.1	1.7	1.5	1.5
Swelling	9.3	1.8	4.6	4.6	4.4	3.9	2.9	2.1	1.5
Abdominal pain	7.4	0.9	5.0	2.2	1.6	1.3	1.2	1.1	1.1
Redness	7.4	1.2	2.5	4.0	4.1	3.4	2.4	1.6	1.0
Itching	7.1	1.2	1.8	2.6	3.2	3.0	2.5	1.8	1.4
Vomiting	2.1	0.2	1.6	0.4	0.2	0.2	0.2	0.2	0.2
Rash	1.9	0.2	0.5	0.6	0.6	0.6	0.6	0.6	0.6
**Any injection site reaction^§^**	60.7	33.1	50.3	41.8	33.1	24.1	15.9	9.7	6.6
**Any systemic reaction^¶^**	76.4	29.8	74.8	44.1	28.9	22.3	19.7	18.2	17.3
Any health impact**	33.9	4.8	33.2	9.7	5.1	3.8	3.3	3.1	3.1
Unable to perform normal daily activities	28.3	3.8	27.7	7.4	4.0	3.0	2.6	2.5	2.5
Unable to work	17.0	1.8	16.3	4.5	2.0	1.3	1.0	1.0	0.9
Needed medical care	1.4	0.1	0.4	0.2	0.3	0.3	0.3	0.4	0.4
Telehealth	0.53	0.02	0.16	0.09	0.11	0.12	0.11	0.12	0.12
Clinic	0.40	0.03	0.05	0.05	0.07	0.10	0.11	0.12	0.12
Emergency visit	0.31	0.04	0.08	0.05	0.06	0.06	0.07	0.07	0.06
Hospitalization	0.04	0.00	0.01	0.01	0.01	0.01	0.01	0.01	0.01

* Proportion of enrollees who reported a reaction or health impact at least once during postvaccination days 0–7.

^†^ Enrollees were able to respond on multiple days.

^§^ Injection site pain, swelling, redness, or itching.

^¶^ Fatigue, headache, myalgia, fever, chills, nausea, diarrhea, abdominal pain, vomiting, or rash at injection site.

** A health impact was defined as being unable to perform normal daily activities, being unable to work, or receiving medical care.

## Discussion

A review of postauthorization safety data after administration of 7.98 million doses of Janssen COVID-19 vaccine during March–April 2021 found that the most commonly reported reactions were similar to those observed in the preauthorization trials (*2*). Among processed VAERS reports, 97% were classified as nonserious events. However, reports included 17 events consistent with TTS, a newly defined condition, including three reports of non-CVST thrombotic events with thrombocytopenia among women aged <60 years during the pause in Janssen vaccine use. Among 88 deaths reported after vaccination, three occurred in patients with CVST (*8*); after preliminary reviews, no other deaths appear to have an association with vaccination.

Two other COVID-19 vaccines, both using an mRNA platform, were authorized for use as a 2-dose series before the Janssen vaccine received authorization. The Janssen adenoviral vector vaccine only requires a single dose for substantial protection from COVID-19 and can be stored at refrigerator temperatures (*2*). Because of these advantages, some health jurisdictions and providers have used the Janssen COVID-19 vaccine among persons for whom ensuring a second dose might be difficult or in settings such as college campuses or drive-through vaccination sites where simple storage requirements are important.^¶¶¶^ In an update of recommendations for use of the Janssen COVID-19 vaccine, ACIP considered the balance between these benefits and a rare but serious safety concern, the risk for thrombosis in large arteries or veins (*10*). On April 23, 2021, after a review of the benefits and risks, ACIP reaffirmed its interim recommendation for use of the Janssen COVID-19 vaccine in all persons aged ≥18 years under the FDA’s EUA (*10*). The EUA now includes a warning for rare clotting events with low platelets, primarily occurring among women aged 18–49 years.

The findings in this report are subject to at least three limitations. First, VAERS data are based on a well-established but passive surveillance system (*9*). Reporting differences are likely, in part because of the EUA requirement that health care providers report all potentially life-threatening events after receipt of the Janssen COVID-19 vaccine. Second, a comprehensive medical review of reported serious adverse events after vaccination, particularly deaths, depends on the availability of medical records, death certificates, and autopsy reports. For many of the serious adverse events reported after vaccination to date, these reviews are in progress. Finally, although v-safe is an important new component of the U.S. COVID-19 vaccine safety monitoring system, participation is contingent on promotion by vaccine administrators and an opt-in enrollment system that uses text messages. Therefore, v-safe data might not be generalizable to the entire population of persons who have received the Janssen COVID-19 vaccine.

The safety profile thus far of the Janssen COVID-19 vaccine is similar to that observed in clinical trials. A rare but serious adverse event occurring primarily in women, blood clots in large vessels accompanied by a low platelet count, was rapidly detected by the U.S. vaccine safety monitoring system. Monitoring for common and rare adverse events after receipt of all COVID-19 vaccines, including the Janssen COVID-19 vaccine, is continuing. Safety data will be evaluated by ACIP as needed to guide benefit-risk assessments of COVID-19 vaccines in use under EUAs.

**Summary**

**What is already known about this topic?**

An Emergency Use Authorization of the Janssen COVID-19 vaccine was granted February 27, 2021. Use was paused during April 12–23, 2021, after detection of six cases of cerebral venous sinus thrombosis (CVST).

**What is added by this report?**

By April 21, nearly 8 million doses of the Janssen COVID-19 vaccine had been administered. Review of safety monitoring data found that 97% of reported reactions after vaccine receipt were nonserious, consistent with preauthorization clinical trials data. Seventeen thrombotic events with thrombocytopenia have been reported, including three non-CVST events.

**What are the implications for public health practice?**

Ongoing monitoring for rare and common adverse events after vaccination is important for evaluating the balance between risks and benefits for each authorized COVID-19 vaccine, including the Janssen COVID-19 vaccine.

## Footnotes

* The Janssen COVID-19 vaccine contains double-stranded DNA encoding a variant of the SARS-CoV-2 spike glycoprotein inserted into a replication-incompetent human adenovirus type 26 virus.

† VAERS reports are classified as serious if any of the following are reported: death, life-threatening illness, hospitalization or prolongation of hospitalization, permanent disability, congenital anomaly, or birth defect.

§ Brighton Collaboration’s draft interim case finding definition for TTS: any patient presenting with acute venous or arterial thrombosis and new onset thrombocytopenia, with no known exposure to heparin or any other underlying condition or explanation for the condition. https://brightoncollaboration.us/wp-content/uploads/2021/04/TTS-Case-Finding-and-Definition-Process.v1.0-1-1.pdf

¶ A health impact was defined as being unable to perform normal daily activities, being unable to work, or receiving medical care.

**
https://vaers.hhs.gov/faq.html

†† Each VAERS report might be assigned more than one MedDRA preferred term. A MedDRA coded event does not indicate a medically confirmed diagnosis. https://www.meddra.org/how-to-use/basics/hierarchy

§§
https://www.cdc.gov/coronavirus/2019-ncov/vaccines/safety/vsafe.html

¶¶ CDC has encouraged jurisdictions receiving COVID-19 vaccines to offer v-safe promotional materials, supplied by CDC, at all vaccination sites. V-safe enrollees receive daily health check-ins via text messages that link to web-based surveys on days 0–7 after vaccination; then weekly through 6 weeks after vaccination; and then 3, 6, and 12 months after vaccination.

*** 45 C.F.R. part 46, 21 C.F.R. part 56; 42 U.S.C. Sect. 241(d); 5 U.S.C. Sect. 552a; 44 U.S.C. Sect. 3501 et seq.

††† Processed VAERS reports are reports that have been coded using MedDRA, have been deduplicated, and have undergone standard quality assurance and quality control review.

§§§ Medical reviews were conducted by CDC and FDA staff physicians, who reviewed all available data, including medical records, death certificates, and autopsy reports, to form preliminary impressions about the cause of death for each decedent. These reviews might be ongoing for some decedents.

¶¶¶
https://doi.org/10.15585/mmwr.mm7018e3
